# Shining a light on Ohno’s dilemma

**DOI:** 10.7554/eLife.99318

**Published:** 2024-06-07

**Authors:** Isabella Tomanek

**Affiliations:** 1 Department of Biology and Department of Biochemistry, University of Oxford Oxford United Kingdom

**Keywords:** Evolution, synthetic biology, ohno's dilemma, adaptive conflict, genetics, *E. coli*

## Abstract

Laboratory experiments on a fluorescent protein in *E. coli* reveal how duplicate genes are rapidly inactivated by mutations during evolution.

**Related research article** Mihajlovic L, Iyengar BR, Baier F, Barbier I, Iwaszkiewicz J, Zoete V, Wagner A, Schaerli Y. 2024. A direct experimental test of Ohno’s hypothesis. *eLife* **13**:RP97216. doi: 10.7554/eLife.97216.

Our cells rely on a large set of genes, each one fulfilling a particular task. But what happens if there is a need for a gene to carry out a new task? Famously, evolution is a tinkerer and rather than evolving new gene functions from scratch, it tends to work with genes that are already doing something similar, albeit poorly (Copley, 2017). All that needs to happen, therefore, is for natural selection to improve upon what is already there.

However, if an existing gene has to perform a new task, it won’t be able to carry out its original role, creating what is known as an ‘adaptive conflict’. In 1970, in his landmark book *Evolution by Gene Duplication*, Susumu Ohno proposed that this conflict could be resolved by having two copies of the same gene: one could do the old task, while the other would be free to evolve towards the new task (Ohno, 1970). Since then, a plethora of sequencing data has revealed the ancestry of a large number of present-day genes that evolved through duplication. However, genomic studies only offer a glimpse into the evolutionary dynamics around the duplication event itself (Kondrashov, 2012).

Now, in eLife, Andreas Wagner (University of Zurich), Yolanda Schaerli (University of Lausanne) and co-workers – including Ljiljana Mihajlovic (Lausanne) as first author – report the results of an experiment that directly tests the hypothesis put forward by Ohno (Mihajlovic et al., 2024). The team experimentally evolved the fluorescent protein coGFP in the bacterium *Escherichia coli;* the bacteria expressed either a single copy of the gene for coGFP or two identical copies. Unlike other fluorescent proteins, coGFP can emit two different colours – blue and green – when stimulated. This allowed Mihajlovic et al. – who are based at Lausanne, Zurich, the Swiss Institute of Bioinformatics, the University of Munster and the Sante Fe Institute – to select for bacteria that emit either green light, blue light, or both.

The gene for coGFP was inserted into *E. coli* using a plasmid (a short circular piece of DNA) that contained either two active copies of the gene, or one active copy and one inactive copy. The duplicate genes were placed so that they faced one another to prevent genetic recombination, as this might lead to further duplications or loss of one gene copy (Andersson and Hughes, 2009; Tomanek et al., 2020). Random mutations were introduced into the genes, and the bacteria that shone the brightest were selected (Figure 1A). This cycle was then repeated multiple times to see whether the bacteria expressing one active gene (the single-copy population) evolved differently to those with two identical copies (the double-copy population).

**Figure 1.**
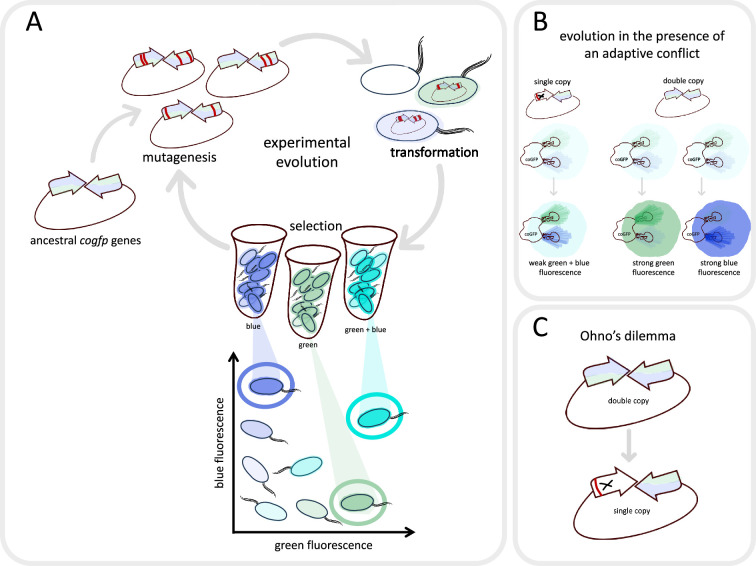
Evolving a fluorescent protein in the laboratory. (**A**) Mihajlovic et al. studied a gene called *cogfp* that codes for a fluorescent protein that can emit both blue and green light. They prepared plasmids containing two active copies of the gene (arrows), and randomly introduced mutations (red lines) into the copies. The plasmids were then inserted into *E coli*. (oval shapes with tails), which were sorted into those that emitted mostly blue light, those that emitted mostly green light, and those that emitted strongly at both wavelengths (represented here as turquoise). The cells which shone the brightest were selected, their plasmids were removed and the cycle was repeated again. (**B**) Mihajlovic et al. carried out the experiment on two populations: bacteria which contained two active copies of the *cogfp* gene (double-copy population; right), and bacteria which contained one active copy and one inactivated copy of *cogfp* (single-copy population; left). According to Ohno’s hypothesis, the single-copy population will experience adaptive conflict: mutations that improve green fluorescence will lead to a reduction in blue fluorescence, and vice versa. Consequently, these bacteria will become only marginally brighter over the course of evolution. In the double-copy population, one gene can evolve to increase green fluorescence while the other can evolve to increase blue fluorescence, resulting in these bacteria becoming significantly brighter over time. (**C**) However, Mihajlovic et al. found that – contrary to what Ohno’s hypothesis would suggest – the increase in brightness was essentially the same for the two populations. This happened because one of the two active genes in the double-copy population had been inactivated (marked with X) by deleterious mutations (red line) during evolution – an effect known as Ohno’s dilemma.

According to Ohno’s hypothesis, if bacteria are evolved using divergent selective pressures (selecting cells which shine brightly blue *and* green), the double-copy population should fare better as the task can be split amongst the two genes (Figure 1B). Yet, Mihajlovic et al. found that both populations evolved increased fluorescence at the same speed. Moreover, when Mihajlovic et al. selected for a single task, green fluorescence, the single-copy population again evolved equally fast as the double-copy population, even though additional gene copies should increase the chance for beneficial mutations to occur (San Millan et al., 2017).

To investigate what was causing this effect, Mihajlovic et al. applied a clever trick of genetic engineering. They placed both copies of the gene for coGFP under the control of different inducible promoters which act like switches that can turn on one fluorescent gene at a time. This revealed that most of the fluorescence detected in the experiments was coming from a single gene, and the other copy had become inactivated by mutations (Figure 1C).

Mutations are like shots being fired at a DNA sequence at random, with most mutations destroying a gene’s function rather than improving it. Mihajlovic et al. found that each gene copy only had a one in four chance of surviving mutagenesis. Consequently, roughly 75% of the bacteria in the single-copy population and roughly 60% of the bacteria in the double-copy population lost fluorescence during the first round of mutagenesis. This effect is the core of what has been termed Ohno’s dilemma (Bergthorsson et al., 2007): if a second gene copy only provides a ‘back-up’ and is not beneficial in itself, it is susceptible to deleterious mutations that inactivate the gene. As such, duplicated genes never stay duplicates for long.

The study by Mihajlovic et al. showcases how researchers can use synthetic biology to dissect evolutionary questions by experimentally constraining mutations. The experimental set-up they created could also be used to test other genes and selective pressures, as well as different hypotheses.

The findings of Mihajlovic et al. suggest that duplicate genes provide redundancy, but these back-up copies are rapidly inactivated by deleterious mutations. However, it is possible that some of the experimental features used when evolving genes in a laboratory (such as high mutation rates and the lack of recombination between the duplicates) may be constraining their evolution. For instance, if recombination, a process that constantly occurs in nature, is allowed to happen between the duplicates, this may lead to a rapid rise in copy number. This transient increase in gene copies could overcome Ohno’s dilemma (Elde et al., 2012; Näsvall et al., 2012), and could play an important role in evolution (Tomanek and Guet, 2022). Maybe letting duplicate genes evolve more freely could teach us more about how nature solves Ohno’s dilemma.
